# A proteomic study to unveil lead toxicity-induced memory impairments invoked by synaptic dysregulation

**DOI:** 10.1016/j.toxrep.2022.07.002

**Published:** 2022-07-07

**Authors:** Nivedha Mohanraj, Neha S. Joshi, Roshni Poulose, Rahul R. Patil, Rashmi Santhoshkumar, Anubhav Kumar, Girish P. Waghmare, Amit Kumar Saha, Syeda Zehra Haider, Yogananda S. Markandeya, Gourav Dey, Laxmi T. Rao, Periyasamy Govindaraj, Bhupesh Mehta

**Affiliations:** a Department of Biophysics, National Institute of Mental Health and Neurosciences (NIMHANS), Bengaluru, India; b Institute of Bioinformatics, Bengaluru, India; c Manipal Academy of Higher Education, Manipal, India; d Electron Microscopy-Common Research Facility, Department of Neuropathology, National Institute of Mental Health and Neurosciences (NIMHANS), Bengaluru, India; e Department of Neurophysiology, National Institute of Mental Health and Neurosciences (NIMHANS), Bengaluru, India; f Centre for DNA Fingerprinting and Diagnostics (CDFD), Hyderabad, India

**Keywords:** Lead toxicity, Synaptosome, Memory, Cognitive impairment, Proteomics, SUMO

## Abstract

Lead (Pb^2+^), a ubiquitously present heavy metal toxin, has various detrimental effects on memory and cognition. However, the molecular processes affected by Pb^2+^ causing structural and functional anomalies are still unclear. To explore this, we employed behavioral and proteomic approaches using rat pups exposed to lead acetate through maternal lactation from postnatal day 0 (P0) until weaning. Behavioral results from three-month-old rats clearly emphasized the early life Pb^2+^ exposure induced impairments in spatial cognition. Further, proteomic analysis of synaptosomal fractions revealed differential alteration of 289 proteins, which shows functional significance in elucidating Pb^2+^ induced physiological changes. Focusing on the association of Small Ubiquitin-like MOdifier (SUMO), a post-translational modification, with Pb^2+^ induced cognitive abnormalities, we identified 45 key SUMO target proteins. The significant downregulation of SUMO target proteins such as metabotropic glutamate receptor 3 (GRM3), glutamate receptor isoforms 2 and 3 (GRIA 2 and GRIA3) and flotilin-1 (FLOT1) indicates SUMOylation at the synapses could contribute to and drive Pb^2+^ induced physiological imbalance. These findings identify SUMOylation as a vital protein modifier with potential roles in hippocampal memory consolidation and regulation of cognition.

**Data availbility:**

The mass spectrometry proteomics data have been deposited to the ProteomeXchange Consortium *via* the PRIDE partner repository with the dataset identifier PXD034212&#34.

## HIghlights

• Early life lead (Pb^2+^) exposure at 100 ppm in rats impairs memory functions.
• Lead exposure at 100 ppm causes dysregulation of the hippocampal synaptic proteome.
• Synaptic SUMOylome could play a major role in Pb^2+^-induced neurodegeneration.

## Graphical Abstract

**Figure:**
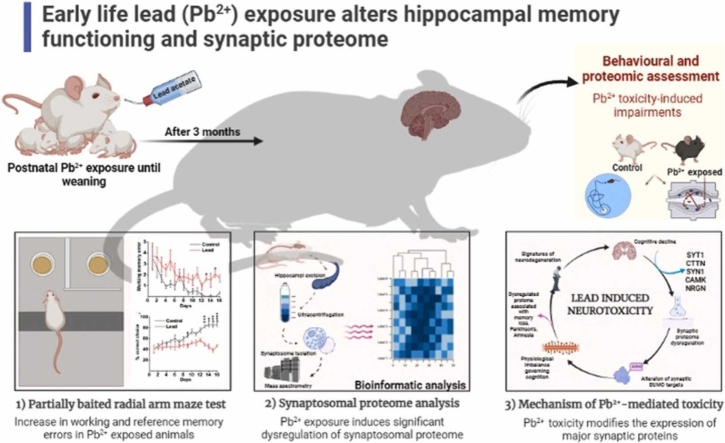

## 1 Introduction

Lead (Pb^2+^) is a heavy metal and is considered a cumulative toxicant because of its chronic side effects on human health. Industrial pollutants, leaded fuels, paints, cosmetics, and vegetables grown in Pb^2+^ polluted soil and contaminated river water are a few of the many sources of Pb^2+^ exposure to humans [1]. The regulations to prevent Pb^2+^ exposure are not strictly followed in the developing countries, predisposing the population to Pb^2+^ exposure *via* drinking water, air, and occupational setup. According to the CDC report, some Indian Ayurvedic and Chinese herbal medicinal preparations were found to have high concentrations of Pb^2+^ [2], [3]. Central Water Commission under the Government of India recently published a report indicating the contamination of major Indian rivers with Pb^2+^ [4]. This poses a severe threat of Pb^2+^ exposure *via* drinking water and agriculture. Exposure of Pb^2+^ from these sources to pregnant mothers affects the overall development of the fetus [5].

Ingestion of Pb^2+^ causes its distribution throughout the body and mainly gets accumulated in the bones. Upon demineralization of bones, Pb^2+^ is slowly released into the bloodstream during pregnancy and lactation [6]. Lead is known to inhibit hematopoiesis, causing anemia, and affecting the kidney and liver functioning [7]. Being a potent central nervous system (CNS) toxin, Pb^2+^ causes cognitive and memory impairments [8]. In addition, Pb^2+^ in the neurons seems to compete with calcium (Ca^2+^) for its binding sites on proteins, thus either mimicking or inhibiting the effects of Ca^2+^ [9]. The hydrated radius of Ca^2+^ and Pb^2+^ is very similar, which might explain why Pb^2+^ acts as a Ca^2+^ agonist and other divalent heavy metals like cobalt, arsenic, and mercury do not [9]. It is also reported that Pb^2+^ can cross the placental barrier and cause developmental defects in the fetus [5]. The population most vulnerable to Pb^2+^ toxicity are children and pregnant and lactating women [6].

Lead, a potent CNS toxin, enhances the risk for cognitive functions in humans. The cellular and molecular mechanisms governing cognition and navigation functions are associated with the hippocampus. During these cognitive processes, dendritic spines undergo structural changes, enlargements, or shrinkage, causing either formation of new synaptic connections or the elimination of the dormant synaptic connections. Such changes may contribute to memory impairments observed in Alzheimer’s disease [10]. Pb^2+^ exposure in the foetal and neonatal phases is reported to cause morphological changes in the dendritic spines in rats [11], [12]. A decrease in dendritic length and spine density is observed in rats heavily exposed to Pb^2+^ [13], which might result in cognitive and memory deficits. Proteins, being the larger part of the cellular machinery, provide a significant functional view of the cells and thereby decide cellular fate. Proteins are a major class of biomolecules and potential drug targets in many diseases [14]. Mass spectrometry-based proteomic analysis has been widely deployed to gain insights into the functional attributes of proteins involved in disease and normal physiology [15]. The mechanism and signalling pathways involved in memory impairment upon Pb^2+^ exposure remain inconclusive. Proteomic analysis of rat hippocampus might unveil dysregulated proteins and thereby enable us to speculate the pathways affected in memory impairment in early life Pb^2+^ exposed rats.

Proteins in the synapse are intricately arranged in huge multimeric complexes through protein-protein interactions. Some of the best characterized synaptic proteins are neurotransmitter vesicle complexes (pre-synaptic) and glutamate receptors-*viz* NMDA, AMPA, and kainate (post-synaptic). The functioning of several of these synaptic proteins is regulated by various post-translational modifications (PTMs) [16]. Among these PTMs, SUMOylation is perhaps the most underexplored protein modification. In SUMOylation, a small ubiquitin-related modifier (SUMO) protein is covalently linked to the target protein [17]. Conjugation of SUMO to the target protein changes its activity, localization, and stability. SUMO proteins are most abundant in the nuclear regions; however, they are also known to show localization in the synapses [18]. A recent study reported the synaptosomal SUMOylome of the developing rat brain and highlighted its importance in synaptic functioning [19]. Intrigued by this information, we decided to explore the synapses of early life Pb^2+^ exposed animals to see toxicity-induced expression changes of proteins that are known to be SUMOylated. A recent study investigated cognitive impairment and oxidative stress in long-term Pb^2+^ exposed rats. Further, the study showed the proteomic profiling of the hippocampus in Pb^2+^ exposed rats and compared it with sham-treated animals [20]. At the single synapse, Pb^2+^ exposure causes memory impairment in rats observed by reduced long-term potentiation (LTP) [21], mirroring the memory deficit observed in behavioural studies [8]. However, there are not enough studies so far to show how chronic Pb^2+^ exposure during lactation causes changes in SUMOylation and in turn in spatial cognition. Hence, the present study attempted to identify potential SUMO targets associated with spatial cognition by performing SUMOylation profiling through postnatal Pb^2+^ exposure in rats. We believe that this is the first study associating potential SUMO targets with Pb^2+^ exposure induced memory deficits in animals.

## 2 Materials

Bradford protein estimation assay kit was procured from Biorad. Osmium tetraoxide, glutaraldehyde and araldite were obtained from TAAB Laboratories Equipment Ltd. Tris base, sodium chloride, disodium hydrogen phosphate and potassium chloride were obtained from SRL Chemical. Sucrose was purchased from G-Biosciences. Sequencing grade trypsin was obtained from Promega and Sep-pak C18 cartridges were purchased from Waters Corporation. Pierce Quantitative Colorimetric Peptide Assay Kit and TMT 10plex labelling kit were procured from Thermo Fisher Scientific. All other chemicals used for the study were purchased from Sigma-Aldrich.

## 3 Methods

### 3.1 Animal experiments and Pb^2+^ exposure

#### 3.1.1 Animal procurement

Healthy female Wistar rats on 18th–19th day of pregnancy were procured from Central Animal Research Facility (CARF), NIMHANS, Bengaluru. Rats were housed in polypropylene cages in a temperature (25 ± 2 °C), humidity (50–55 %), and light (12-h light–dark cycle) controlled environment with food and water provided *ad libitum*. All the experiments were conducted according to the ethical guidelines of the Committee for the Purpose of Control and Supervision of Experiments on Animals (CPCSEA) and approved by the Institutional Animal Ethics Committee (AEC/70/460/B.P., 08–08–2019) of NIMHANS, Bengaluru. All efforts were made during the study to minimize suffering and the number of animals utilized.

#### 3.1.2 Lead exposure in rats

**Fig. 1:**
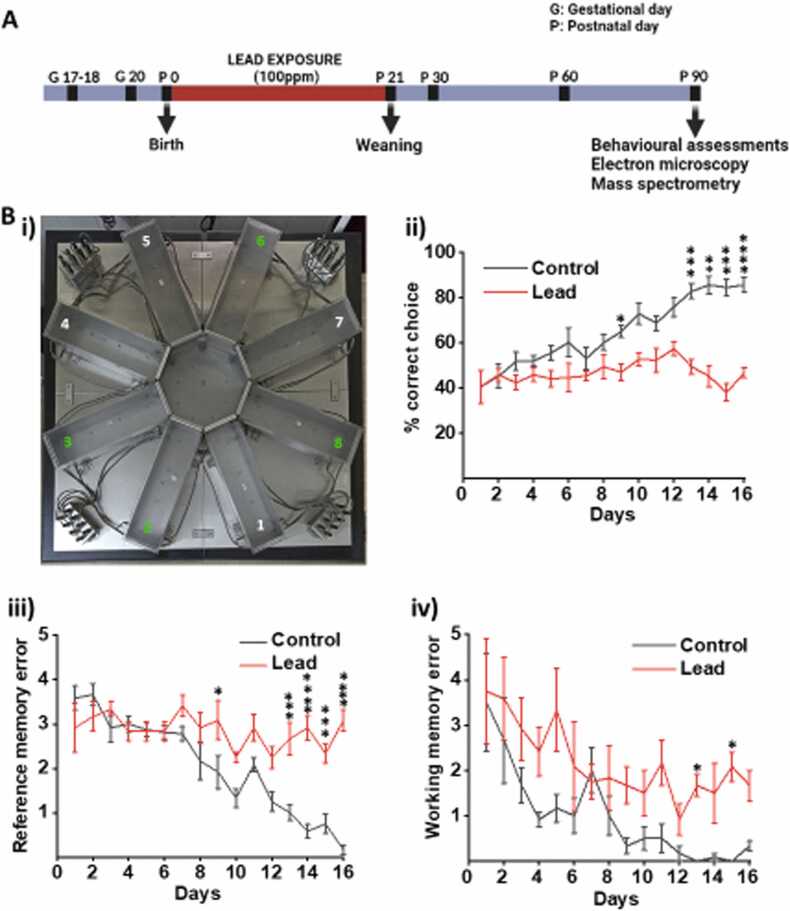
The detrimental effect of neonatal exposure of lead on the memory. (A) Experimental design abstract. (B. i) Partially baited 8 arm radial maze with the baited arms (2, 3, 6, and 8 marked green), (B. ii) Percentage of correct choices, (B. iii) Reference memory error, and (B. iv) working memory error in the lead exposed animals as compared to the controls. Data is expressed as mean±SEM. ****p < 0.0001, ***p < 0.001, **p < 0.01, *p < 0.05, two-way ANOVA. n = 6 rats/group.

Pregnant female rats post-delivery were administered 100 ppm of lead acetate in drinking water (Fig. 1) [22], [23]. On day 21 after birth, the litters were weaned with no more exposure of Pb^2+^ from the mother’s milk. During this period, litter size, body weight and water intake were monitored. Sham control animals (henceforth written as control) were not exposed to lead acetate. Both the control and the early life Pb^2+^ exposed rats were used for experimentation upon attaining an age of 3 months.

#### 3.1.3 Partially baited radial arm maze test (PBRAM)

For the behaviour analysis, 6 control and 6 Pb^2+^ exposed animals were used. Each of the animals were habituated in the maze for 10 min for 3 days, where food pellets were placed in all eight arms. During RAM training, bait was kept semi-randomly in only four selected arms and same was followed for all rats ensuring similar spatial relations between bait positions and distal spatio-visual cues for all the animals. Training was conducted until all the rats achieved a cross learning criteria of 80 % correct choices which took upto 14–16 days. Experiments were carried out between 8 a.m. and 3 p.m every day. In the daily sessions, rats were placed individually on the central platform facing different directions, and they were allowed to orient themselves. All the animals were permitted to choose among the different arms until they completed the session by either entering all the baited arms or until 5 min had elapsed, whichever was first. The maze was thoroughly cleaned of all cues and animal droppings after each rat with 70 % alcohol. A partially baited radial arm maze allows the estimation of percent correct choice, reference and working memory errors simultaneously. The behavioural parameters measured included (a) total number of correct choices into baited arms (percent correct choice), (b) total number of entries into unbaited arms (reference memory error, RME) and (c) total number of re-entries to either baited or unbaited arms (working memory error, WME).

#### 3.1.4 Synaptosome preparation

Hippocampal synaptosomes were isolated from adult Wistar rats (200–250 g) as described earlier [24], [25] with minor modifications. Synaptosomal isolation was carried out from 5 sets of control and 5 Pb^2+^ exposed animals each (each set was obtained by pooling hippocampi from 5 control and 5 Pb^2+^ exposed animals). Briefly, animals were anesthetized using isoflurane, decapitated and the brains were quickly removed and placed in ice cold phosphate buffered saline (PBS) (in mM: 137 NaCl, 2.7 KCl, 8 Na_2_HPO_4_, and 2 KH_2_PO_4_; pH 7.4). Hippocampi were collected and homogenized in 10 volumes of ice-cold Tris-Sucrose buffer (in mM: 20 Tris-base, 320 Sucrose; pH 7.4; supplemented with protease inhibitor cocktail). The homogenate was then centrifuged at 2300xg at 4° C for 10 min to remove cellular debris and nuclear fraction. The supernatant from this low-speed spin was collected and centrifuged for 45 min at 200,000xg and 4° C using an ultracentrifuge (OPTIMA XPN90, Beckman Coulter). The pellet was re-suspended in 2 ml of Tris-Sucrose buffer and layered onto 4 ml of 1.2 M sucrose solution and centrifuged for 30 min at 230,000xg and 4° C. The gradient interface was then removed and layered onto 2 vol of 0.8 M sucrose solution and centrifuged for 30 min at 230,000xg and 4° C. The resulting pellet was re-suspended in Tris-Sucrose buffer and stored at −80° C. The protein concentration was determined using Bradford protein estimation assay. A total of five synaptosomal isolations each from the control and Pb^2+^ treatment groups were taken for mass spectrometry.

#### 3.1.5 Transmission electron microscopy (TEM)

Fresh synaptosome pellets were immersed in 3 % buffered glutaraldehyde and postfixed in 1 % buffered osmium tetroxide. Following post fixation, the pellet was then dehydrated using graded series of alcohol (70 %, 80 %, 90 % and 100 %) and embedded into pure resin. Ultrathin sections (70 nm) were taken using a Leica UC6 ultramicrotome. The sections were collected on copper grids and contrasted using saturated methanolic uranyl acetate and 0.2 % lead citrate and examined under JEM – 1400Plus, JEOL transmission electron microscope (TEM).

### 3.2 Proteomic sample preparation

#### 3.2.1 Protein digestion

Equal amount (~250 µg) of protein from all the animal preparations were taken for further processing. Proteins were reduced using 5 mM Dithiotheritol for 30 min at 60 °C, which was followed by alkylation with 10 mM Iodoacetamide and incubated in the dark for 30 min at room temperature. Reduced and alkylated samples were subjected to protein precipitation by adding 5 volumes of chilled acetone and incubated at −20 °C overnight. These were then centrifuged at 5000 x *g* for 5 min to pellet down the protein. Pellet was resuspended in 50 mM triethyl ammonium bicarbonate buffer (TEABC) and subsequent protein digestion was carried out using sequencing grade trypsin at 1:20 ratio (enzyme: protein) at 37 °C for 12–14 h. Samples were acidified with 1 % formic acid and peptides were cleaned using Sep-pak C18 cartridges, later vacuum dried and stored until further use.

#### 3.2.2 TMT-labelling

Peptide estimation was carried out using Pierce Quantitative Colorimetric Peptide Assay Kit. TMT labelling was performed according to the instructions of the manufacturer. Briefly, the TMT-10-plex was used for labelling, and labels were reconstituted in 100 µL of anhydrous acetonitrile. The dried trypsin digested peptide preparations were reconstituted in 100 µL of 50 mM TEABC (pH 8.0). TMT labels were added to the appropriate samples, and the reaction mixture was incubated for 1 h at room temperature. After incubation, the reaction was quenched with 8 µL of 5 % hydroxylamine. Samples were pooled, dried, and processed further for fractionation.

#### 3.2.3 Basic reverse phase liquid chromatography (bRPLC)

The dried pooled peptide sample was reconstituted in 5 mM ammonium formate buffer and fractionated into 96 fractions using bRPLC. The fractions were pooled further into 12 fractions, dried and cleaned using in-house C18 stage tips. Eluent was dried in speed vac and analyzed on a mass spectrometer.

#### 3.2.4 Liquid chromatography with tandem mass spectrometry (LC-MS/MS)

LC-MS/MS data acquisition was carried out on an Orbitrap Fusion mass spectrometer (Thermo Electron, Bremen, Germany), interfaced with Easy-nLC1000 nanoflow LC system (Thermo Scientific, Odense, Denmark). All fractions were analyzed into technical triplicates. The peptides were later reconstituted in 0.1 % formic acid and loaded onto a trap column (nanoviper 2 cm, 3 µm magic C18Aq, Thermo Scientific). Peptides were resolved on an analytical column (nanoviper 25 cm (75 µm silica capillary, 3 µm magic C18, Thermo Scientific)), at a flow rate of 300 nL/min, using a linear gradient of 2–38 % solvent B (0.1 % formic acid in 100 % ACN) for 100 min. The total run time was 120 min. Data-dependent acquisition with full scans in 350–1600 *m/z* range was performed using an Orbitrap mass analyzer at a mass resolution of 120,000 at 400 *m/z*. The most intense precursor ions (top 20) from a survey scan were selected for MS/MS fragmentation using higher energy collision dissociation (HCD) fragmentation, with 34 % normalized collision energy and detected at a mass resolution of 50,000. AGC target value was set to 1,000,000 with maximum ion injection time of 150 ms.

#### 3.2.5 Data analysis

The raw data acquired from mass spectrometry was searched using SEQUEST search algorithm against Rat RefSeq database using Proteome Discoverer version 2.1 (Thermo Fisher Scientific, Bremen, Germany). The search parameters included carbamidomethylation of cysteine residues (+57.02 Da), TMT modification at peptide N-terminus and lysine side chain as a fixed modification, oxidation of methionine (+15.99 Da) and peptide N-terminal acetylation (+42.01 Da) as dynamic modifications. MS/MS spectra were searched with a precursor mass tolerance of 10 ppm and a fragment mass tolerance of 0.05 Da. Trypsin was specified as the protease with a permission of maximum of two missed cleavages. The data was searched against target decoy database, and the false discovery rate was set to 1 % at the peptide level.

#### 3.2.6 Statistical and bioinformatic analysis

For behavioural data, statistical analysis was performed by two-way ANOVA using Origin Pro software. Statistical analysis for the quantified proteins was carried out using the Perseus software platform. Two sample t-test was done, and the p-value ≤0.05 was set as a cut-off for significant proteins. The fold change was calculated, and 1.2-fold change cut-off was applied for significantly dysregulated proteins from control to Pb^2+^ exposed animals. Gene ontology (GO) of differentially expressed proteins was carried out using the DAVID GO tool available online (https://david.ncifcrf.gov/). In addition, ToppGene analysis was performed in order to understand the correlation of dysregulated proteins with human diseases (https://toppgene.cchmc.org/).

## 4 Results

### 4.1 Early life Pb^2+^ exposure causes memory deficits in rats

Research in the recent past on chronic occupational lead exposure leading to learning and memory associated disabilities [8] and on developmental Pb^2+^ exposure in rats showed impairments in short-term and long-term memory [20]. This highlights the need to analyze the mechanism of Pb^2+^ toxicity. In the present study, we subjected the animals to radial arm maze tests determining critical parameters associated with spatial cognition to study the effect of early life Pb^2+^ exposure on memory formation.

Behavioural experiments carried out in Wistar rats exposed to Pb^2+^ through lactating mother until weaning (21 days post-birth) showed significant memory deficits. Memory impairment in the adult rats was assessed by evaluating spatial learning index, working memory error (WME), and spatial memory error (SME) for the control and Pb^2+^ exposed groups. The control animals reached the criteria of above 80 % correct choice on day 13 (p < 0.0001) in the radial arm maze (Fig. 1B i), and it continued till the end of the task (day 16), while the Pb^2+^ exposed group of rats failed to reach the criteria and their performance declined after 13 days and reached 46 % at day 16 (Fig. 1B ii). Animal entries into arms 1, 4, 5, and 7 in the eight-arm radial arm maze were considered as reference memory errors (RMEs) in 16 days trial. Pb^2+^ exposed rats showed a significant impairment in reference memory. The number of RMEs increased significantly in Pb^2+^ exposed animals when compared to control animals as indicated by RME mean of 0.16 (+/-) and 3.08 (+/-) in control and Pb^2+^ exposed animals, respectively (Fig. 1B iii). Working memory errors represent the repetitive entries in baited and unbaited arms in PBRAM. Working memory impairment was assessed using the 16 day PBRAM task. The statistical analysis using Student’s t-test revealed no significant differences in WME in the Pb^2+^ exposed animals compared to the control animals on day 1 until day 9, but showed significant difference (p < 0.05) on day 13 and 15. In terms of number of correct choices, Pb^2+^ group animals showed decline on day 10, day 13–16 when compared to control group which was statistically significant (p < 0.05). (Fig. 1B iv). Overall, the Pb^2+^ exposed animals showed a significant decline in memory-related learning tasks compared to the control group as observed from changes in percent correct choices and RME.

### 4.2 Transmission electron micrographs confirm the quality of the synaptosomal preparations

**Fig. 2:**
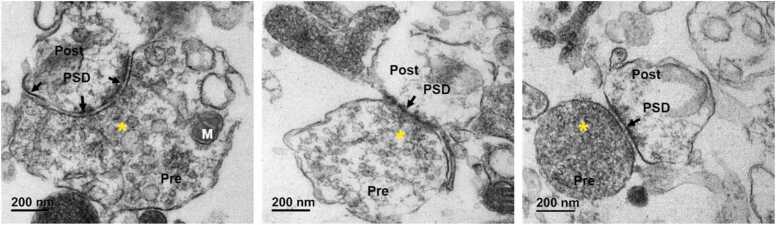
Representative transmission electron micrographs of synaptosome preparation showing characteristic tightly apposed pre-synaptic (Pre) and post-synaptic (Post) elements. Electron dense post-synaptic densities (PSD, arrow) can be seen under the post-synaptic membrane. The pre-synaptic bouton is filled with numerous synaptic vesicles (SVs, asterisk) and mitochondria (M). Scale bar represents 200 nm. (Magnification: x15000).

Gestational lead exposure is previously reported to reduce presynaptic glutamate release by altering NMDA receptor expression [21] and affects levels of cognition-related proteins such as presynaptic synaptosome-associated protein-25 (SNAP-25) and postsynaptic density protein-95 (PSD-95) [26]. To study the effect of Pb^2+^ exposure on the hippocampal synapse and understand the mechanism of Pb^2+^ induced decline in memory functioning (Fig. 1), we performed the proteomic analysis of the synaptosomal fractions from the two experimental groups. Therefore, synaptosomal fractions isolated from the rat hippocampi were assessed for the quality of the preparation by transmission electron microscopy prior to mass spectrometric analysis. Ultrathin sections of synaptosomal pellets from control post-fixation and processing from control animals were analyzed to identify synaptic components. The data revealed a sufficiently enriched population of intact synaptosomes with pre-synaptic elements (Pre; asterisks indicate pre-synaptic boutons with vesicles) and multiple post-synaptic densities (PSD; arrows) (Fig. 2).

### 4.3 Lead exposure alters hippocampal synaptic proteome

**Fig. 3:**
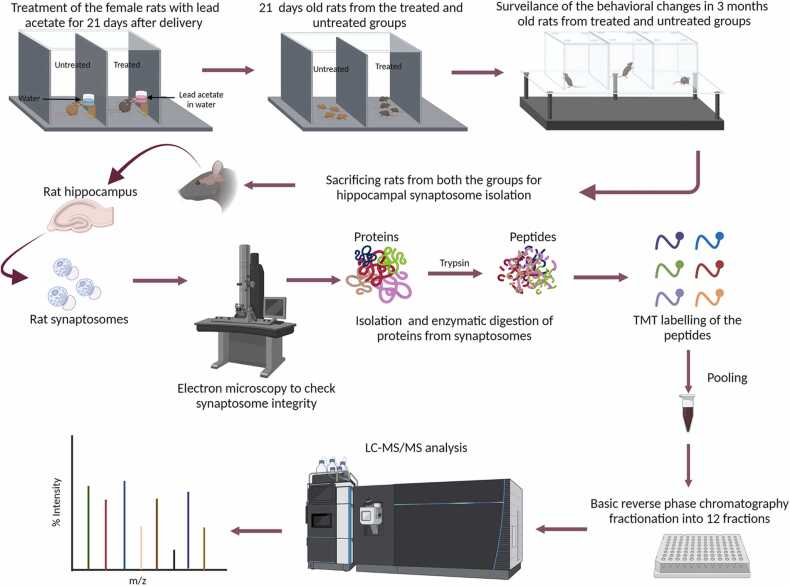
Overall workflow of the proteomic study carried out for synaptosomal proteome profiling.

Following mass spectrometry, proteomic profiling was performed by searching the raw data against the Rat RefSeq database using Proteome Discoverer 2.1. We have taken five independent synaptosome preparations from control and Pb^2+^ exposed groups for the proteomic analysis. The overview of the proteomic analysis of the rat synaptosomes is depicted in Fig. 3. A total of 7285 proteins were identified, (Supp. Table S1) of which 6894 proteins were quantified in the data. Around 5700 proteins with high confidence and supported with at least two unique peptides and PSMs were further considered for statistical and bioinformatics analysis (Supp. Table S2). Statistical analysis of the 5700 proteins revealed 1076 proteins as p-value significant (p ≤ 0.05). A fold change cut-off of 1.2 when applied to the p-value significant data resulted in 289 proteins that were dysregulated in the Pb^2+^ exposed group as compared to the control group. Of these, 180 were upregulated while 109 proteins were downregulated in the Pb^2+^ exposed group (Supp. Table S4).

### 4.4 Gene ontology of the synaptosomal proteome divulged various functional aspects of Pb^2+^ exposure in rats

**Fig. 4:**
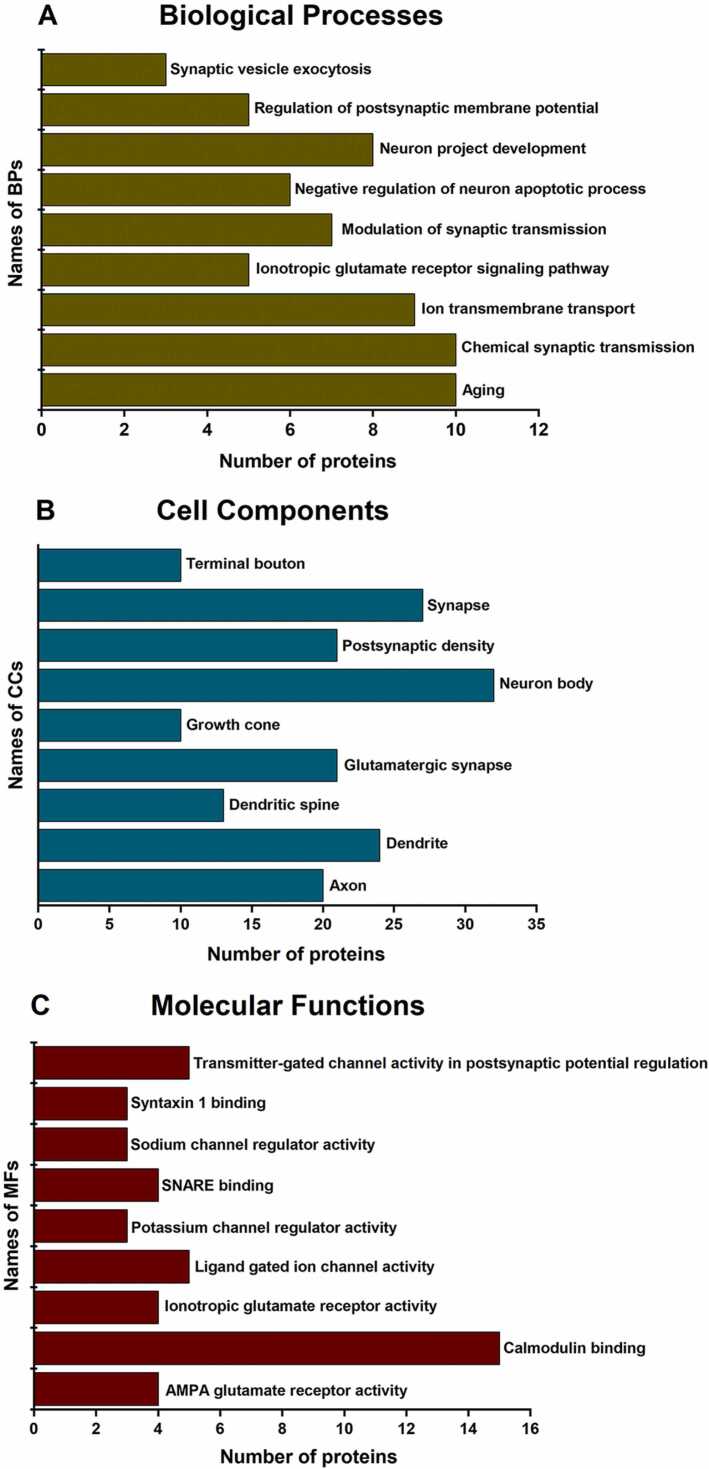
Bar graphs representing the number of proteins identified from Gene Ontology analysis associated with A) biological processes (BPs), B) cell components (CCs) and C) molecular functions (MFs).

**Fig. 5:**
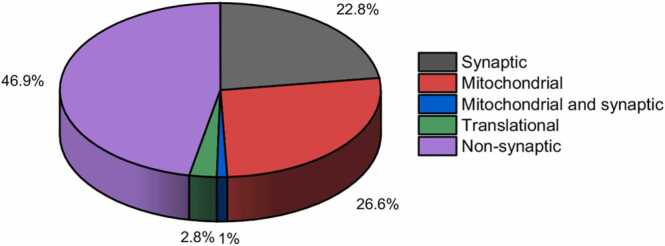
Pie chart showing the classification of the various significantly dysregulated proteins (289) among the control and lead exposed groups.

Maternal Pb^2+^ exposure is previously reported to decrease the expression of brain development and cognition-related proteins [26]. To investigate if Pb^2+^ exposure induced memory decline is due to its effect on synaptic structure and function, we performed gene ontology (GO) enrichment analysis of the synaptic proteins (5700 proteins) using DAVID for biological processes (BPs), cellular components (CCs), and molecular functions (MFs). Based on which, we picked enriched proteins associated with synapses across the biological systems for analysis. A total of 5438 proteins were identified by the DAVID database search. Interestingly, this included a large number of proteins from BPs which showed significant association with regulation of axogenesis, dendritic spine morphogenesis, dendritic spine maintenance, endocytosis and exocytosis, learning and memory, long term synaptic potentiation and depression, neurotransmitter uptake and release, synaptic transmission, *etc* (Fig. 4A, Supp. Table S11). Conforming to the enriched synaptosomal preparation, CCs indicated a large number of proteins classified with synapse, dendrite, neuron spine, pre/post-synapse, postsynaptic density, synaptic membrane, SNARE complex, synaptic vesicle membrane, *etc* (Fig. 4B, Supp. Table S12). Further corroborating our hypothesis, MFs showed various proteins categorized as ion channel-binding, voltage-gated Ca^2+^ channel activity, GABA- and glutamate receptor-binding, neurotransmitter-binding, SNARE- and syntaxin-binding, *etc* (Fig. 4C, Supp. Table S13). In addition, some of the other major pathways from the GO enrichment analysis included translational initiation, mitochondrial electron transport and ATP synthase, *etc*. showing many proteins localized to the mitochondria and translation initiation complex. The analysis of proteins of interest (POIs) targeting neuron-related processes from BP, CC, and MF hits using DAVID identified a unique list of 5452 proteins. This showed that 95 % (5452/5700) of the identified proteins have known functions associated with processes affecting neuronal physiology and functioning. As mentioned earlier, a total of 1076 p-value significant proteins were identified from the Perseus analysis of high confidence proteins, (p < 0.05; Supp. Table S3). On comparison with POIs, a total of 592 proteins were identified conforming to synaptic dysregulation in early life Pb^2+^ exposure. Interestingly, we also identified mitochondrial and translation-related proteins as significantly dysregulated among the two groups (Fig. 5, Supp. Table S5). Of these dysregulated proteins, GO analysis indicated 164 POIs demonstrating association with memory functions (Supp. Table S7), which were further analyzed for their functional relationship with Pb^2+^ exposure.

### 4.5 Modulation of synaptic proteins by SUMOylation regulates memory

**Fig. 6:**
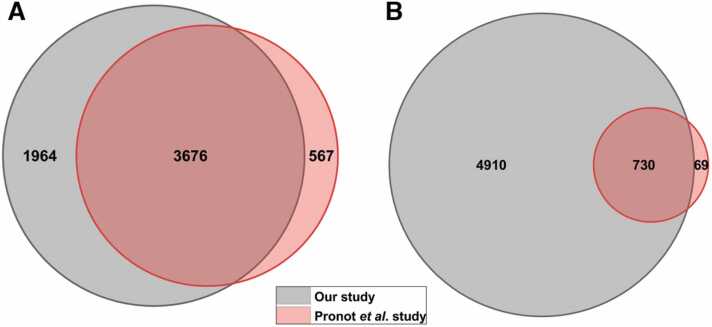
Venn diagrams representing the synaptosomal proteomes from the current study and Pronot *et al.*’s study. (A) Venn diagram depicting the total synaptosomal proteome (identified in both control and lead treated groups) in the present study and Pronot *et al*.’s study. (B) Venn diagram showing synaptosomal SUMOylome published by Pronot *et al*. and potential SUMO targets identified in the present study.

Recent studies indicate the role of synapse specific SUMOylation in various states of neurodevelopment and neurodegeneration [27]. To analyze the relevance of synaptic SUMOylome in the hippocampus of the Pb^2+^ exposed group, we probed into the SUMOylome and SUMO target proteins identified from the analysis. Therefore, to understand the role of SUMOylation in cognition and neurodegeneration, we compared our rat hippocampal synaptosomal proteome with the recently published rat forebrain synaptosomal proteome. Pronot *et al*. have previously reported 4379 synaptosomal proteins from the rat forebrain, which they used as a reference synaptic proteome. This represented more than 88 % of the proteins found in at least three previously published synaptic proteomes [19]. In the present study, we identified 5700 proteins (high confidence, PSM/Unique Peptides > 2) in the rat hippocampal synaptosomes. On comparison, we observed 86 % overlap with the data published by Pronot *et al*. and an additional 1964 proteins were found unique to our database (Fig. 6A, Supp. Table S8). On closer examination by SUMO enrichment analysis, Pronot *et al*. identified 803 SUMO2/3ylated proteins. A comparison between synaptosomal proteome from our data and the SUMOylome from Pronot *et al*. revealed 730 proteins (90 %) as potential SUMO targets from the present study (Fig. 6B, Supp. Table S9). This represents 12 % of our total synaptosomal proteome. The list of total synaptosomal proteins identified in the present study largely overlapped with the previously characterized synaptosomal protein datasets and the synaptic SUMOylome discussed in Pronot *et al*.

On comparison with the array of dysregulated proteins previously identified, we found 45 proteins that were SUMO targets. Interestingly, of these, 32 SUMO-specific proteins had functions that could be possibly corroborated with synaptic dysfunction observed in Pb^2+^ toxicity. Previously, based on bioinformatics analysis we observed synaptosome enriched proteins associated with aging, chemical synaptic transmission, modulation of synaptic transmission, regulation of long-term potentiation, synapse organization, exocytosis, *etc*. localized to the synapse, dendritic spine, terminal bouton, synaptic vesicle, *etc*. Therefore, these results emphasize the importance of studying the role of synaptic SUMOylome in synaptic organization, functioning, plasticity and transmission.

### 4.6 Lead toxicity leads to differential regulation of potential target proteins involved in synapse organization and functioning

**Fig. 7:**
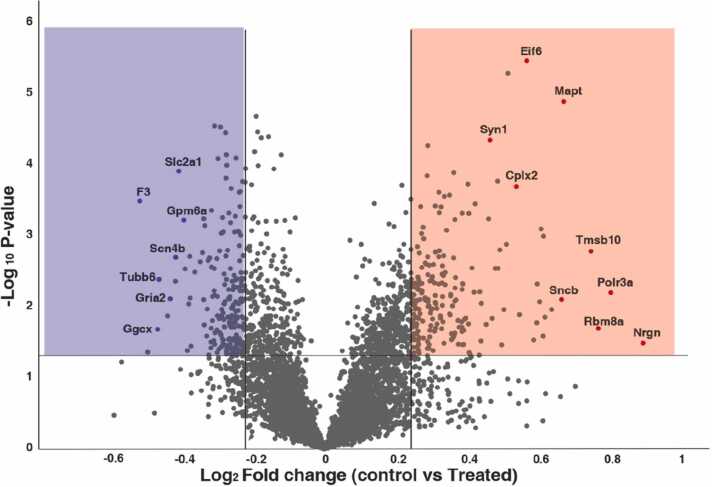
Volcano plot showing the representation and distribution of proteomic data from rat synaptosomes. Highlighted in red are the proteins overexpressed in synaptosomal fractions from rats treated with lead. The proteins which show downregulation are highlighted in blue.

Various studies speculated the pre- and post-synaptic targets of Pb^2+^ [9], [28], however an in-depth analysis of the synaptosomal proteome associated with hippocampal memory formation was overdue. A recent study pointed out that low-level gestational Pb^2+^ exposure in rats showed reduced dendritic spine plasticity with a decline in learning and memory [12]. In this section, we identify potential proteins that could govern synapse function alterations observed because of Pb^2+^ induced toxicity. We identified a set of target proteins that could significantly govern the phenotype observed with Pb^2+^ toxicity. Further, we observed various significantly dysregulated proteins involved in ionotropic glutamate receptor signalling, synaptic transmission, neuron projection regulation, neurotransmitter regulation, synapse organization, synaptic vesicle exocytosis, *etc*. These proteins are listed in Table 1 with their functions and fold change values, which could explain the Pb^2+^ exposure-associated cognitive impairments we observed in our experimental group. In addition, volcano plots were generated to show the distribution of proteins, highlighting overexpressed proteins in red and the downregulated proteins in blue. Proteins are significantly dysregulated and separated by a horizontal line which shows the p-value cut-off of 0.05 (Fig. 7). Some of the functionally important proteins identified in the present study that were differentially regulated among control and Pb^2+^ exposed groups are listed in Table 1**.**

**Table 1:** List of functionally important synaptic proteins identified in our study showing significant dysregulation upon Pb2+ exposure.

S. No.	Protein name	Function	Fold change	References
1	Synatotagmin 1 (SYT1)	Major synaptic vesicular protein that acts as a principal Ca2 + sensor	0.61	[33], [34]
2	Src substrate cortactin (CTTN)	Involved in receptor-mediated endocytosis *via* clathrin-coated pits	2.27	[35]
3	Nedd4 binding protein 3 (N4BP3)	Functions associated with axon and dendrite arborization during cranial nerve development	2.3	[36]
4	Acid sensing ion channel 2 (ASIC2)	A cation channel with high affinity to Na^+^ and permeable to Li^+^ and K^+^	2.68	[37]
5	Synapsin-1 isoform a (SYN1)	A neuronal phosphor protein that regulates neurotransmitter release	1.37	[38]
6	Ca+2/calmodulin-dependent protein kinase II gamma (CAMK2G) Ca^2+^/calmodulin-dependent protein kinase type II subunit delta (CAMK2D)	Belong to the family of Ca^2+^/calmodulin-dependent protein kinase could play a crucial role in regulating synaptic transmission and LTP maintenance	0.54 0.57	[39]
7	Annexin6 isoform (ANXA6)	A Ca^2+^ dependent membrane binding protein that regulates release of Ca^2+^ from intracellular stores	0.57	[40]
8	Neurogranin (NRGN)	A postsynaptic protein kinase C substrate which might play a role in regulation of long term potentiation and chemical synaptic transmission	1.86	[41], [42]

Some of the identified SUMO targets of our interest which were significantly dysregulated from GO analysis include glutamate ionotropic receptor AMPA type subunits 2 and 3 (GRIA2 and GRIA3), which play important roles in excitatory synaptic neurotransmission (FC = 0.57 and 0.62 respectively) [29], [30]. On further analysis, we identified a few proteins which showed significant differences (p < 0.05) between control and Pb^2+^ exposed samples although they weren’t highly dysregulated.

These proteins listed in Table 2 are key targets of SUMOylation in the synapse and could have potential roles in synaptic neurotransmission and affect neuronal morphogenesis, functioning and physiology. In addition, other potential SUMO targets that were significantly regulated between the two conditions included mitochondrial proteins such as apoptosis-inducing factor 1, mitochondrial (AIFM1), and glutathione S-transferase kappa 1 (GSTK1), and ubiquinone biosynthesis monooxygenase (COQ6). Recently, a few studies have indicated the role of SUMOylation in regulating mitochondrial homeostasis [31], and SUMOylation mediated regulation of mitochondrial function is also implicated in Parkinson’s disease [32]. It would be interesting to study how Pb^2+^ affects mitochondrial dynamics and the association of SUMOylation targets in regulating it.

**Table 2:** List of synaptic SUMO target proteins identified among Pb2+ exposure and control groups.

S. No.	Protein name	Function	References
1	Metabotropic glutamate receptor 3 (GRM3)	Modulates spontaneous epileptiform activity in the granule cells in DG region of hippocampus	[43]
2	Synapsin 3 (SYN3)	Plays role in regulation of neurotransmitter release and initial axon outgrowth	[44]
3	Lysosome-associated membrane glycoprotein 5 (LAMP5)	Tunes GABAergic synaptic transmission, thus affecting short term synaptic plasticity	[45]
4	Ionotropic glutamate receptor subtype NMDA 1 ((GluN1), GRIN1)	A subunit of NMDAR, provides a glycine binding site to the receptor	[46]
5	Double C2-like domain-containing protein alpha (Doc2-alpha, DOC2A)	Sensor of neurotransmission, helps in the regulation of neurotransmitter release	[47]
6	Flotillin-2 (FLOT2)	Regulates axonal regeneration and growth	[48]
7	Gamma-aminobutyric acid type B receptor subunit 2 (GABA-B receptor 2, GABBR2)	Essential for signal transduction by GABA-B receptors	[49]
8	Solute carrier family 12 member 5 (Electroneutral potassium-chloride cotransporter 2, SLC12A5)	A potassium-chloride co- transporter KCC2 encoded by SLC12A5, with fundamental roles in mediating fast synaptic inhibition by maintaining a hyperpolarizing gradient for chloride ions	[50]
9	SLIT-ROBO Rho GTPase-activating protein 2 (SRGAP2)	SRGAP2 co-regulates the density of spines and the length of the spine neck.	[51]
10	Tumor protein p63-regulated gene 1-like protein (TPRG1)	A vertebrate-specific presynaptic phosphoprotein interacting with Bassoon and synaptic vesicles (SVs) with unknown functions, also called Mover	[52]

**Fig. 8:**
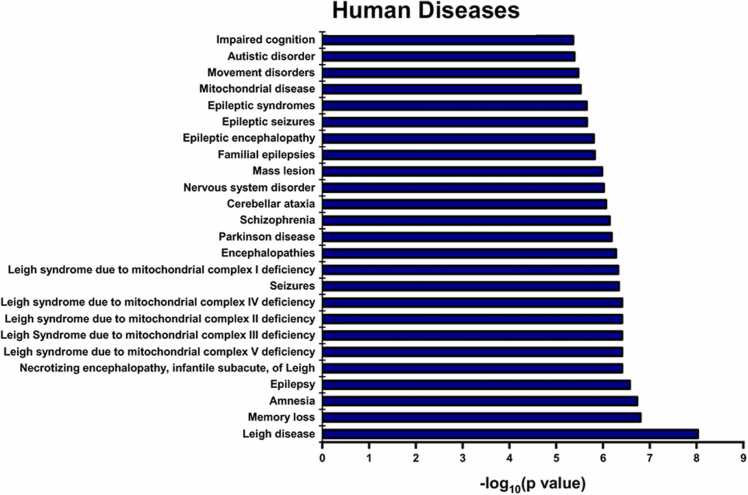
Human disease enrichment analysis using ToppGene for significantly dysregulated proteins showing the top 25 hits. Represented as –log (p value).

Furthermore, we performed ToppGene analysis to compare the dysregulated proteins with human disorders. Out of the 289 dysregulated proteins, 277 proteins were mapped to various human diseases by the tool. The top 25 hits based on p-values displayed proteins that segregated with memory loss, amnesia, epilepsy, Schizophrenia, Parkinson’s, nervous system disorder, *etc*. Interestingly, the analysis also showed various mitochondrial diseases such as Leigh syndrome due to mitochondrial complex I-V deficiencies, alteration in NADH dehydrogenase [ubiquinone] flavoprotein 3 expression (NDUFV3), providing more reasons to investigate Pb^2+^ toxicity on mitochondrial physiology as stated earlier (Fig. 8, Supp. Table S10).

## 5 Discussion

Even though mankind has been well aware of the adverse effects of Pb^2+^ since ancient times [53], scientists worldwide majorly focussed on its impact on learning and memory [8], while the mechanism of Pb^2+^ toxicity still remains unclear. In an attempt to verify these reports, our study demonstrated the significant impact of Pb^2+^ toxicity on spatial learning and memory with deficits showing an increase in reference and working memory errors (Fig. 1B). Furthermore, proteomic analysis of the synaptosomal fraction showed differential regulation after early life Pb^2+^ exposure.

### 5.1 Effect of Pb^2+^ in the pre-synapse

In the presynaptic terminal, action potential induced increase in cytosolic calcium (Ca^2+^) from extracellular or intracellular stores (*i.e.*, ER and mitochondria) activates Ca^2+^ binding proteins, leading to neurotransmitter release into the synaptic cleft. As Pb^2+^ mimics Ca^2+^ in the cell, it affects all the proteins which are regulated by Ca^2+^. Lead present in the cytosol binds to calmodulin with a higher affinity than Ca^2+^ [54]. Binding of Pb^2+^ to calmodulin activates Cam kinase II which phosphorylates synapsin 1, causing its dissociation from the synaptic vesicles fusing with the plasma membrane. Interestingly, both Cam kinase II and synapsin 1 were found to be significantly dysregulated in the present study. Synaptotagmins are Ca^2+^ sensors and are affected by Pb^2+^, as it binds to the C_2_A and C_2_B domains of synaptotagmins. Additionally, the conversion of evoked potential to neurotransmitter release is mediated by synaptotagmins [55], [56], [57]. In our proteomics study, synaptotagmin 1, one of the dominant synaptotagmins in the brain, was found to be significantly downregulated. This might explain how intracellular Pb^2+^ decreases neurotransmission caused by evoked potentials as reported by Manalis *et al.* [58]. Synaptotagmin 7, which mediates asynchronous neurotransmitter release [59], and synaptotagmin 3 were also found to be downregulated in the present study. Moreover, the effect of Pb^2+^ on Ca^2+^ mediated exocytosis is also evident from its effects on annexins. Annexins are a class of Ca^2+^ regulated proteins that help in docking two negatively charged membrane surfaces [60], [61]. Annexins form a complex with S100 proteins forming dimers, facilitating membrane docking and also play an important role in endocytosis [60]. Interestingly, Annexin A6 and S100A13 were found to be downregulated in the present study, suggesting a probable mechanism of perturbation of exocytosis rate by Pb^2+^. Lead is also known to block channel activity as it passes through voltage-dependent Ca^2+^ channels in the bovine adrenal medulla [62], possibly explained by similar hydration radii of the two ions. In this study, many of the voltage-dependent L-type Ca^2+^ channel subunits such as CACNB4, CACNA2D1, CACNA2D3, and CACNA1C showed significant differences between the two groups, however, they did not qualify the fold change (FC) cut off for differential regulation (Supp. Table S3). Besides, it is quite interesting to note that proteins like V-type proton ATPase 116 kDa subunit a1 (ATP6V01A), which acidifies vacuoles [63], and NEDD4 binding protein (N4BP3), which aids in axon and dendrite arborization [64], were found to be upregulated in the present study. It could be possible that the overexpression of these proteins activates compensatory mechanisms to counter the loss of synapses and neuronal death resulting from Pb^2+^ toxicity.

### 5.2 Effect of Pb^2+^ in the post-synapse

Various glutamate receptors such as GRIA1, GRIA2, GRIA3, GRIA4, GRIN1, GRM1, GRM2, GRM3, GRM4, GRM7, and GRM8 were downregulated in the present study. Glutamate, the most common neurotransmitter, binds to glutamate receptors (ionotropic and metabotropic) on the post-synaptic membrane. As fewer neurotransmitters are released in the synaptic cleft because of Pb^2+^ toxicity, a smaller number of these receptors are engaged with the neurotransmitters. With the downregulation of glutamate receptor expression on Pb^2+^ exposure, the probability of glutamate—receptor interaction is further reduced, thus significantly reducing the strength of EPSPs [65]. Lead, being a divalent ion, interacts with the Zn^+^ binding sites [66] or maybe on a different site [21] in the NR1 subunit blocking the flow of Ca^2+^ ions and also inhibiting NMDARs. Although, AMPA and Kainate receptors were unaffected by the presence of Pb^2+^ in the cell. However, the receptors for GABA such as GABBR2, GABRA2, GABRA4, GABRA5, GABRB1, GABRD, and GABRG2, which is an inhibitory neurotransmitter, were found to be downregulated in the present study. This indicated that Pb^2+^ toxicity could affect the IPSPs generated in the postsynaptic neuron. NOTCH 1, a single-pass transmembrane receptor regulating memory and synaptic plasticity [67], [68], was found to be significantly downregulated in this study (FC 0.63). It is previously reported that loss of NOTCH 1 function in a mouse model displayed impairments in synaptic plasticity and memory [69]. NOTCH1 is essential for the functioning of NMDAR, as it interacts with the NR1 subunit of NMDAR, and loss of *Notch 1* results in lowered expression of NR1 subunit [70]. Consistent with these reports, both NOTCH 1 and GRIN1 (a type of NMDAR) are downregulated in the present study. Further, NOTCH 1 also regulates ERK and CREB activation by regulating the expression of CamKII [70]. Our results endorse this data, as CamKII shows a significant decrease in expression in Pb^2+^ exposed animals.

Lead exposure to the rat offspring through lactation also causes oxidative stress in the neurons [26] leading to a decrease in neuronal pH [71], which might acidify the synaptic cleft. Under such conditions, non-voltage-gated Na^+^ channels present on the postsynaptic membrane are transiently opened. These channels are known as Acid-sensing ion channels (ASICs) [37]. Evidently, ASIC2 was found to be significantly upregulated (FC 2.6) in the present study. Opening of these channels can significantly increase the intracellular concentration of Ca^2+^ in the post-synaptic neuron and might also cause Ca^2+^-mediated cytotoxicity [37]. In addition, neurogranin (NRGN) a postsynaptic protein expressed exclusively in the brain (cortex, striatum & hippocampus) concentrated in the dendritic spines [72], was significantly upregulated in the present study. Neurogranin, associated with PSDs, plays a vital role in synaptic plasticity and long-term potentiation to enhance memory and cognition [73], also known to be downregulated in dementia patients [74]. On the contrary, NRGN was found to be upregulated (FC 1.86) in the present study. As the Pb^2+^ exposure in our study was only for 21 days, it is possible that upregulation of NRGN is a compensatory mechanism to maintain memory and cognition, which could also be evidenced by a study with Mild Cognition Impairment (MCI) patients showing elevated levels of neurogranin in their CSF samples [75]. Furthermore, cortactin (CTTN), a postsynaptic linker protein plays a crucial role in actin stabilization which maintains the shape of dendritic spines [76] and elevates plasma membrane NMDAR [21], was also found to be highly upregulated in the present study. As it is previously established, Pb^2+^ toxicity causes loss of dendritic spines [11] and reduction in NMDAR expression [28]. Therefore, overexpression of CTTN also seems to be an attempt to maintain synaptic morphology and plasticity.

### 5.3 SUMOylation of the synaptic proteins

The complex synaptic organization is maintained by ‘300,000’ proteins that work in symphony to carry out an intricate neurotransmission process [77]. These proteins and their functions are directed by many post-translational modifications (*viz* phosphorylation, acetylation, methylation, ubiquitination, *etc*.). However, currently, the most novel and least explored is SUMOylation, a reversible and transient PTM capable of altering protein localization in the cell [78]. Intending to explore its crucial functions in the synapse, we compared our synaptosomal proteome with the SUMOylome published by Pronot *et. al.* [19]. On comparison, we discovered essential proteins implicated in the process of neurotransmission were SUMO targets in our proteome. Proteins directly related to synaptic plasticity, like CTTN, CAMKV, FLOT1, and FLOT2*,* many glutamate receptors, and DOC2A were found to be SUMO targets. Some other vital synaptic proteins which were not found in Pronot *et. al.* SUMOylome were also SUMO targets, like SYT1 [79], SYN1 [80], and SNCA [81]. This likely suggests that SUMOylation is a major player in modulating synaptic plasticity. As Pb^2+^ affects synaptic plasticity, SUMOylation might play a critical role in the phenotype observed in early life exposure of Pb^2+^. Its function in neurotransmission cannot be ignored and further deeper investigation needs to be carried out to explore their role in governing synaptic plasticity.

An earlier study through molecular and behavioural analysis in rats identified SYT1 as a synaptic protein involved in age related loss in hippocampus-associated memory and learning [82]. It is reported that SYT1 expression is reduced in Alzheimer’s disease brains as a result of synaptic loss [83]. Cortactin, localized in pre- and post-synaptic structures, recently when studied in knockout mice showed deficits in hippocampus-dependent spatial memory formation with impaired long-term potentiation and complete loss of structural spine plasticity [84]. Noteworthy, another study discusses the implications of mutations in Parkin, a ubiquitin ligase, causing Parkinson’s disease. Interestingly, the study identified CTTN as a key regulator involved in the clearance of impaired mitochondria, a major process associated with suppression of Parkinsonism [85]. In addition, SUMOylation has been previously linked to various brain disorders such as Alzheimer’s *via* its role in regulating long-term potentiation and hippocampal-dependent learning [86] and Parkinson’s by interfering with mitochondrial dynamics [32]. These reports are in conjunction with our findings from disease annotation, possibly correlating the mechanism of Pb^2+^ toxicity by means of pathways affected in various neurodegenerative disorders some of which could be mediated by SUMOylation. This opens up the avenue of exploring biological markers and therapeutic targets of Pb^2+^ and other brain disorders.

## 6 Conclusions

Lead exposure to pups through lactation represents the imminent danger faced by working mothers as a result of lenient regulations on Pb^2+^ contamination, a major industrial health hazard, increasing the vulnerability of their progeny to heavy metal toxicity. A combination of behavioural and proteomic studies revealed downregulation of key presynaptic and postsynaptic proteins directly governing processes such as synaptic transmission, calcium homeostasis, and synaptic plasticity. This data corroborates with our findings suggesting deleterious effects of early life Pb^2+^ exposure on learning and memory. Additionally, various compensatory mechanisms are also set into motion because of this toxicity which could explain the high expression of proteins such as CTTN, NRGN, and SYN1, which are of major interest in synaptic release, long term potentiation, and neurotransmission. Further, SUMOylation, with recent studies pointing towards its role in regulating various physiological processes at the synapse, could serve as a vital checkpoint in understanding the molecular machinery of Pb^2+^ induced neurodegeneration. Moreover, our results also shed light on dysregulated proteins such as SYT1, CTTN, and other SUMO target proteins which are implicated in various states of neurodegeneration, providing an avenue for the study to pave way for therapeutic strategies. Hence, it is of utmost importance to meticulously investigate the lethal effects of Pb^2+^ poisoning on the health of all living beings and the ecosystem. We believe this study sets the trail highlighting the need to intensely explore the avenue of PTMs affecting synapse organization in neurodegeneration.

## Declaration of Competing Interest

The authors declare that they have no known competing financial interests or personal relationships that could have appeared to influence the work reported in this paper.
